# Update on the surgical management of Graves’ orbitopathy

**DOI:** 10.3389/fendo.2022.1080204

**Published:** 2023-02-06

**Authors:** Joonyoung Baeg, Han Sol Choi, Charm Kim, Hyuna Kim, Sun Young Jang

**Affiliations:** ^1^ Department of Ophthalmology, Soonchunhyang University Bucheon Hospital, Soonchunhyang University College of Medicine, Bucheon, Republic of Korea; ^2^ Department of Ophthalmology, AIN Woman`s Hospital, Incheon, Republic of Korea; ^3^ Department of Ophthalmology, Soonchunhyang University Seoul Hospital, Soonchunhyang University College of Medicine, Seoul, Republic of Korea

**Keywords:** surgery, Graves’ orbitopathy (GO), decompression, strabismus, LID

## Abstract

Graves’ orbitopathy (GO) is a complex autoimmune disorder of the orbit that causes the eye to appear disfigured. GO is typically associated with Graves’ disease, an inflammatory autoimmune condition that is caused by thyrotropin receptor autoantibodies. Although our knowledge of the pathophysiology of GO has improved, its exact pathogenesis remains unclear. Some patients suffer from disfigurement, double vision, and even vision loss rather than hyperthyroidism. The disease severity and activity prompt different treatments, as the signs of GO are heterogeneous, so their management can be very complex. Despite medical advances, the first-line treatment for moderate-to-severe active GO is still glucocorticoids, while surgery can be critical for the treatment of chronic inactive GO. Surgery is sometimes required in the acute phase of the disease when there is an immediate risk to vision, such as in dysthyroid optic neuropathy. Most surgeries for GO are rehabilitative and subdivided into three categories: decompression, strabismus repair, and lid surgery. This review is a basic overview of the field, with up-to-date knowledge of the surgical techniques for GO. We review and summarize recent literature on the advances in surgery for GO to provide up-to-date insights on the optimal surgical treatment for GO.

## Introduction

Graves’ disease (GD) is an autoimmune disease of the thyroid gland; autoantibodies bind to the thyrotropin receptor on thyroid follicular cells. The annual incidence of GD is estimated to be 20-30 per 100,000 according to studies in Swedish populations (1–3) and approximately 20-50 per 100,000 according to more recent reviews (4, 5). The disease principally affects women aged 30 to 40 years, and the overall prevalence is 0.5% (6). Graves’ orbitopathy (GO) is a complex inflammatory disorder of the orbit typically associated with GD. Limited data are available regarding GO incidence (7). A recent review clearly summarized what is known (7). According to Bartley (1994), the age-adjusted incidences of GO were 16 and 2.9 per 100,000 person-years in women and men, respectively (8). A recent Danish study investigated the nationwide incidence of thyroid eye disease (TED), a synonym of GO (9). The mean annual nationwide incidences of TED were 8.0 and 1.9 per 100,000 person-years in women and men, respectively; the mean incidence was 5.0 in the overall population, which included both women and men. Notably, GO develops in up to 50% of patients with GD (10–14). In a Swedish study, 75% of hyperthyroid patients had GD; 20% of these patients had thyroid-associated eye symptoms/signs (3). However, radiological orbital imaging revealed subtle abnormalities in 70% of patients with GD, although the patients reported no symptoms (15). The overall prevalence of GO is unclear. In 2013, a large Italian study reported that 73.7% of GD patients exhibited no ocular involvement, whereas > 20% of GD patients experienced mild, moderate-to-severe, and sight-threatening GO (16).

Most GO patients respond to conservative treatment and do not require surgery. However, approximately 5% of GO patients undergo surgery in the first year after diagnosis; up to 20% of GO patients undergo surgery within the first decade (17). When GO develops into dysthyroid optic neuropathy (DON), surgical intervention is required. In a British population, DON patients constituted approximately 2% of all GO patients; they initially received steroids, but nearly 50% of the patients required surgical orbital decompression within 9 months (18). In 2008, a nationwide cohort study in Denmark revealed that the incidence of diplopia in GO patients was approximately 17-51% (19). The 4-year cumulative incidence of strabismus was 10%, and 8% of such cases required surgery (9). A multidisciplinary approach combining medical and surgical strategies may benefit GO patients. Because signs of GO are heterogeneous, management can be complex. The European Group on Graves’ orbitopathy (EUGOGO) recently stated that an optimal treatment has not been identified (20).

Typically, GO can be categorized into two phases: active and inactive. Active GO is associated with a progressive active inflammation over 6-24 months that expands the extraocular muscles (EOMs) and orbital fat. The presence of edema, inflammation, and accumulated interstitial glycosaminoglycan lead to the expansion of orbital contents. The condition can subsequently develop into proptosis; conjunctival chemosis; upper eyelid swelling; hyperemia of the eyelid, conjunctiva, and/or plica; strabismus; and (most seriously) a corneal ulcer or DON. When severe, the orbital apex can become crowded by the expansion of orbital soft tissues. These changes can trigger DON, a sight-threatening complication experienced by up to 4-8% of GO patients (8, 21, 22). A multicenter study regarding the clinical features of DON in Europe showed that orbital imaging could reveal apical muscle crowding in 88% of DON patients (23). However, the authors of the study noted that DON patients may lack severe proptosis and orbital inflammation (23). Most active GO is assumed to be self-limiting because, unlike the target organs of other human autoimmune diseases (e.g., the synovium in rheumatoid arthritis), most of the orbit lacks lymphoid tissue. Thus, lymphoid neogenesis may not occur within the orbit, which limits the duration of the autoimmune disease (24). However, the autoimmune process involves incapacitating sequelae that are usually initially active but then inactive (12, 25). Some patients experience disfigurement, double vision, and vision loss, rather than hyperthyroidism. Furthermore, in addition to causing daily physical discomfort, GO symptoms negatively impact mental health (26) and quality-of-life (27). Among patients with mild GO, 15-20% experience progression to greater severity, as reflected by a change in the clinical activity score (24). Laurberg et al. (28) reported that approximately 5% of GD patients developed moderate-to-severe GO; it was unclear whether their definition of moderate-to-severe GO included sight-threatening GO. Nevertheless, approximately 2-5% of patients with GO will progress to moderate-to-severe disease (13, 29–32). When sight-threatening GO is absent, but symptoms are seriously disabling in terms of significantly compromising daily life, EUGOGO recommends immunosuppression (if GO is active) and surgical intervention (if GO is inactive). Patients with moderate-to-severe GO usually have two or more of the following: lid retraction ≥ 2  mm, moderate or severe soft tissue involvement, exophthalmos ≥ 3  mm above the normal ethnicity- and sex-specific value, and constant or intermittent diplopia (33, 34). A small minority of patients require surgery when the self-limiting inflammatory phase has passed.

Thus far, first-line treatment to reduce orbital inflammation has involved high-dose glucocorticoids. In patients with moderate-to-severe GO, intravenous glucocorticoids are more effective and cause fewer adverse effects, compared with oral glucocorticoids (20). Immunosuppressants (azathioprine, cyclosporin) and orbital irradiation can be combined with oral or intravenous glucocorticoids as second-line treatments for moderate-to-severe GO (20). Recently, teprotumumab, a 150-kDa monoclonal antibody against IGF-1R, was reported to be effective and safe in patients with moderate-to-severe GO; the drug provides proptosis improvement similar to the result of orbital decompression surgery (35, 36). Nevertheless, surgery plays an important role in the treatment of chronic inactive GO. Importantly, surgery is rarely performed in the acute phase, which may involve DON, despite clinical evidence that early orbital decompression can limit progression to more severe disease in patients with significant orbital congestion (24). According to the 2021 EUGOGO guidelines, among patients with active GO, orbital decompression is indicated for patients with severe exposure keratopathy, DON patients who do not respond to intravenous glucocorticoids within 1-2 weeks, and patients with recent eyeball subluxation (20). Most surgery is rehabilitative and can be subdivided into decompression, strabismus repair, and lid surgery. Generally, orbital decompression surgery is usually performed first, followed by strabismus surgery and then lid surgery (37, 38), because orbital decompression surgery may affect strabismus status. Additionally, decompression and/or strabismus surgery can affect the contours and/or heights of the upper and lower eyelids (39).

Overall, the surgical GO options are sparse, and the chosen method is determined on the basis of specific changes to the orbit. The procedure of choice when correcting globe proptosis is orbital wall decompression; squint surgery is often used to treat persistent diplopia. This surgery seeks to preserve binocular single vision in both the primary and downgaze positions; patients are thus likely to exhibit residual diplopia in other gaze directions (40). Blepharoplasty lowers the eyelids, lifts the midface, and reduces the brow fat pad; this surgery removes bags and tightens the skin. Upper lid retraction can be treated via levator advancement or Mueller muscle recession surgery (41).

This paper summarizes current knowledge regarding GO surgery; we systematically reviewed literature in the PubMed database. Thus, this is a basic overview of GO surgery, with up-to-date insights concerning optimal surgical treatments.

## Orbital decompression

Orbital decompression is widely presumed to improve exophthalmos and DON. Orbital decompression may involve only fat removal; alternatively, it may involve one-, two-, three-, or four-wall bone decompression with or without orbital fat removal. The procedure is performed using an external or an endoscopic approach. Surgical orbital decompression involves the removal of fat and/or one or more of the bony walls to provide space for overgrown EOMs and orbital fat tissue. Although many studies have been conducted regarding orbital decompression, no consensus has emerged with respect to an optimal approach. Considering the absence of randomized controlled trials, no procedure is considered better than others. Additionally, a synthesis is difficult because studies vary in terms of surgeon expertise, patient disease stage, surgical indications, and evaluation methods (42).

In 1911, Dollinger was the first to introduce orbital decompression with lateral wall decompression (43). Subsequently, the Walsh-Ogura technique was established for use in removing the ocular floor and medial orbital wall (44). Orbital decompression for GO management has further evolved over the past 30 years; several approaches have been combined to simultaneously remove multiple walls, and an endoscopic approach has been introduced in conjunction with a navigation system. Recently, the surgical indications for proptosis have expanded to include esthetic improvement. The mean decrease in the Hertel exophthalmometric value after surgery was 4.56 mm (45). Furthermore, GO activity and severity were alleviated by orbital decompression, but the clinical activity score and the modified NOSPECS [No physical signs or symptoms, Only signs, Soft tissue involvement, Proptosis, Extraocular muscle involvement, Corneal involvement and Sight loss (due to optic nerve compression)] classification were associated with significant postoperative decline (46). Visual acuity significantly improved in DON patients who underwent decompression surgery, and postoperative visual acuity increased in 82-88% of such patients (47).

After fat decompression alone, the short/intermediate-term and long-term decreases were 4.2 mm and 5.9 mm, respectively (48). A few reports have indicated that orbital fat removal is safer than, but as effective as, bone wall decompression (49–51). When fat and bone are simultaneously removed, fat removal generally leads to 2-4 mm of reduction in ocular protrusion (47). However, one study revealed that although fat removal increased the effectiveness of surgery, statistical significance was only attained for three-wall decompression (42). Fat decompression was associated with a limited incidence of new-onset diplopia, and cerebrospinal fluid leakage was not reported, in contrast to the findings after lateral wall decompression (48). Periocular fat removal relieved intraorbital pressure and was effective in DON patients (52–54). Medial orbital wall decompression effectively treated mild-to-moderate exophthalmos accompanied by diplopia (17). Several studies showed that medial decompression provided a mean postoperative Hertel difference of 4.36 mm (45). Various surgical methods have been used to approach the medial wall, including transantral, transcutaneous, transconjunctival, intranasal, and transcaruncular routes (55–59). Notably, the transcaruncular approach enables optimal exposure and safe access to the medial periosteal space. In DON patients with mild ocular protrusions, the transcaruncular approach is recommended to relieve optic nerve compression. The medial orbit wall can also be accessed using an endoscopic nasal approach, thereby improving operative performance and safety when engaging in posterior decompression (47). The combination of medial and inferior wall decompressions enables a slightly greater reduction of 4 to 6 mm (47, 60, 61). However, this approach may be associated with marginally greater rates of regression and new-onset double vision, compared with balanced two-wall decompression (17). An endoscopic approach to the orbital wall was first described by Kennedy et al. in the early 1990s (58). Studies in recent decades have shown that endoscopy can regress exophthalmos by 3.2-4.7 mm (62). Further recession is possible with the addition of an external approach, such as lateral orbital wall removal (62). Although the endoscopic approach did not improve protrusion to the degree achieved using the transconjunctival approach, the postoperative diplopia rate was lower with the endoscopic approach (42).

Lateral orbital wall decompression, first described by Kronlein in the late 19th century, remains a common method for treatment of GO (63). Generally, single lateral wall decompression is preferred when treating exophthalmos with a moderate protrusion (3-7 mm) (64). The effectiveness of such an approach in DON patients remains controversial; the posterior effect may be less than the effect of medial wall decompression in terms of decompressing the orbital apex. However, lateral wall decompression reduces proptosis by 2.7-4.8 mm (45, 65–67). Recently, deep lateral decompression has become more popular (45, 67) because it provides satisfactory decompression with minimal complications, along with the potential for use in combination with other techniques (e.g., medial wall decompression and fat removal) (68–71). The removal of thin bone above the temporalis muscle can trigger some complications. Specifically, medial movement of the temporalis muscle may cause the muscle to occupy the newly decompressed space, thus displacing soft orbital soft tissue back into the orbit. One study indicated that the use of polyethylene-coated titanium implants may be promote sidewall decompression (72). The postoperative diplopia rate after deep lateral decompression is 0-8.6% (67). Other complications include dry eye syndrome, vibration, temporal hollowing, rectus muscle injury, cerebrospinal fluid leakage, and hemorrhage (17, 64, 67). Balanced decompression (i.e., concurrent removal of medial and lateral walls) is recommended for patients with severe proptosis who do not exhibit diplopia (17). Balanced decompression may significantly improve mild-to-moderate proptosis in patients lacking diplopia. The mean proptosis reduction was 3.1 to 5.6 mm (45, 73–75); this reduction was statistically significant, and the extent of reduction was greater in double-wall groups than in single-wall groups (76). Proptosis reduction was more evident in patients with higher preoperative Hertel values; significantly lower reductions were apparent in patients with less preoperative proptosis (75). Balanced decompression may be maximally effective; it is safe in terms of causing minimal complications (61, 77–79).

For patients with severe exophthalmos, three-wall decompression is preferred (47). Because more extensive wall removal improves ocular proptosis, such decompression minimizes the orbital symptoms. A few nonrandomized studies have revealed that three-wall decompression may maximally improve exophthalmos, but it carries an increased risk of complications (61, 80–83). Decompression of the medial, inferior, and lateral walls considerably reduced ocular protrusion by 4.5-7.5 mm (45, 74, 79, 84). The mean reduction was significantly greater after three-wall decompression than after two-wall decompression, although three-wall decompression is most effective for patients with more severe preoperative exophthalmos (42). After three-wall decompression, new-onset diplopia and orbital complications are not uncommon. The results of multiple (non-controlled) descriptive studies have suggested that although three-wall decompression most effectively improves exophthalmos, it increases the rates of complications (mainly diplopia and hypoglobus) (61, 85, 86). Notably, three-wall decompression maximally reduced ocular proptosis and normalized the Hertel values, even in extreme cases (75). Some clinicians suggest that avoidance of post-orbital diplopia should not be the only goal; it is important to achieve normal Hertel values and eye symmetry. If three-wall decompression does not relieve DON, orbital roof decompression can be considered (47). Another option is four-wall decompression, which constitutes an extreme form of decompression. This complex procedure can trigger brain herniation via the orbital roof opening, thereby reducing the orbital volume associated with a pulsatile eye or eyeball (45). This technique is not recommended unless the exophthalmos is extremely severe. Recently, image-guided navigation has been used during orbital decompression surgery to further improve pronounced exophthalmos. This approach reduces the operating time, as well as the incidences of postoperative complications (e.g., diplopia and strabismus) (87, 88). The types of decompression are summarized in **Table 1**.

**Table 1:** Types of decompression.

	Decompressed wall	Indication	Effect	Features
Only fat	None	Mild to moderate	4.2 to 5.9 mm	· Effective in DON patients, relieves IOP. · Complications usually include diplopia. · When combined with bony decompression, fat removal improves protrusion by 2 to 4 mm.
Single wall (med.)	Med	Mild to moderate	4.36 mm	· Transcaruncular approach is generally recommended · Endoscopic approach remains available
Single wall (lat.)	Lat	Moderate	2.7 to 4.8 mm	· Easily accompanied with med. wall decompression · Effect on DON is controversial
Two-Wall (med. & inf.)	Med. & Lat.	Moderate	4 to 6 mm (3.2 to 4.7 mm for endoscopic approach)	· Ext. approach - more effective improvement · Endoscopic approach - lesser postoperative diplopia · Higher rate of postoperative diplopia compared to balanced two -wall decompression
Balanced Two-wall	Med. & Lat.	Mild to moderate or severe	3.1 to 5.6 mm	· Usually effective and safe; leads to fewer complications.
Three-Wall	Med., Lat., Inf.	Severe	4.5 to 7.5 mm	· Most effective in terms of improving protrusion, but leads to higher rate of postoperative complications (e.g., diplopia and hypoglobus).
Four-Wall	Med, Lat., Inf., Sup.	Severe	More effective than three-wall decompression	· Usually not recommended unless exophthalmos is extremely severe or three-wall decompression has failed.

Few papers have adequately reported the rates of orbital complications. Leong et al. stated that the global incidence of complications was 9.3%, whereas the global incidence of serious complications with long-term sequelae was 0.12% (89). Diplopia is the most common postoperative complication of decompression surgery. The primary cause of diplopia after such surgery is EOM misalignment (90). After surgery, patients with preoperative diplopia were more likely to experience primary gaze diplopia regardless of the surgical technique used; thus, it was essential to preoperatively measure any primary positional misalignment (81). New-onset postoperative diplopia developed in a mean of 18-29% patients (64, 91); however, after fat decompression alone, the proportion was as low as 3.3% (48). Medial wall decompression triggered diplopia in 0-35% of patients (56, 60, 61, 64, 82), whereas the rate after lateral wall decompression was 0-6% (70, 92–95). New-onset diplopia was less common after lateral decompression than after other types of bone decompression (64). One study showed that lateral decompression did not increase the risk of diplopia, but bilateral surgery did (96). The incidences of diplopia after medial and inferior wall resection could reach 50% (97). Patients who undergo balanced medial and lateral wall decompressions may experience shifts in the symmetrical medial and lateral rectus values, theoretically reducing the risk of diplopia (98). However, several studies have revealed diplopia rates of 10-20% (61, 81). One study demonstrated that balanced decompression increased the risk of new-onset diplopia (96). It has been suggested patients who underwent balanced medial and lateral wall decompression surgery were likely to have shifted symmetric medial and lateral rectus, which in theory reduces the risk of postoperative diplopia (98). However, in several studies the incidence rate of diplopia is reported to be in the range of 10-20% (61, 81). One study found balanced decompression increased the risk of developing new diplopia after surgery (96). The “balancing effect” of sidewall and medial wall decompressions may limit diplopia (78, 99, 100), although some studies have shown no change in the incidence of diplopia (101, 102). The highest diplopia rate was observed after three-wall decompression of the medial, medial inferior, and lateral walls (75). In several studies, three-wall decompression was associated with a 14-57% incidence of diplopia (17, 64). Some researchers have suggested that the association between lateral orbital wall decompression and postoperative strabismus is weaker than the associations of other wall decompressions with postoperative strabismus (103). One nonrandomized retrospective study compared lateral orbital wall decompression to balanced medial and lateral wall decompression; it revealed a relationship between lateral wall removal and a higher resolution rate in patients with preoperative strabismus (78).

The facial numbness rate after lateral surgery is 24%, but the numbness is generally mild and transient (< 3 months) (64). Lateral decompression tends to exhibit an association with a higher rate of postoperative numbness, compared with other types of bone decompression, but the differences are not statistically significant (64). In one study, numbness was recorded in 35% of 98 patients who underwent lateral wall decompression surgery; it persisted in 14% of those patients for 2 years (104). Other adverse effects include transient or permanent paresthesia of the suborbital nerve area in approximately 24% of patients (74), as well as immediate periorbital ecchymosis and edema, postoperative bleeding and infection, corneal erosion, sinusitis, cerebrospinal fluid leakage, abscesses, hematomas, and acute subdural hemorrhages (64, 74, 94, 105). The most common complications of endoscopic procedures are sinusitis, frontal or maxillary mucus production, cerebrospinal fluid fistular leakage, nasolacrimal duct lesions, strabismus, and diplopia. Strabismus may spontaneously disappear within 3-4 weeks but reappear during subsequent disease progression; correction is then necessary (62). Vision loss is rare after various orbital surgeries, including tumor resection, post-traumatic reconstruction, and GO decompression (42, 106). Vision loss can be triggered by tissue expansion and compression by a hematoma, the onset of hypotension while under general anesthesia, vasospasm, and/or the onset of optic nerve mechanical damage/ischemia caused by arterial occlusion (42). Among the many types of orbital surgery, orbital GO decompression exhibits the lowest risk of vision loss (42, 106); the prevalence ranges from 0.09% to 0.52% (73, 107, 108). One study showed that orbital decompression surgery for GO patients triggered a significant decline in retinal nerve fiber layer thickness (109). Most patients undergo a single orbital decompression procedure; the reoperation rate after first decompression ranged from 1.7% to 13.8% (110). Thus, there is minimal literature concerning repeat orbital decompression. The reasons for reoperation include persistent protrusion and/or optic neuropathy, as well as recurring optic neuropathy with a GO flare (110). Reoperation status has been associated with younger age, normal thyroid function, high-level preoperative orbital protrusion, and preoperative steroid treatment (111).

Many of the studies mentioned above were nonrandomized, retrospective case series; thus, the results are not directly comparable. No evidence-based conclusions can be drawn regarding an optimal decompression procedure (i.e., the procedure with the lowest complication rate). Well-planned, prospective/longitudinal, randomized clinical trials are required to compare the surgical methods used for orbital decompression of GO patients. Such trials would yield reliable empirical evidence.

## Squint surgery

During the late phase of GO inflammation, orbital fibrotic changes tighten the EOMs and thus restrict their movements (112). The most commonly affected muscle is the inferior rectus (IR; the bulkiest and most tonically active muscle), followed by the medial rectus (MR) and superior rectus (SR) (113). Affected muscles exhibit enlarged bellies on computed tomography; the tendons are typically spared (114).

Observation only is recommended when a patient lacks symptoms of diplopia in the primary gaze or the reading position. In such situations, conservative treatment options (e.g., Botox or a Fresnel prism) may aid acute-phase patients and patients with small deviations. Because the IR muscle is most commonly affected, restricted motility triggers binocular diplopia and advanced upgaze positioning. Most patients adopt chin-up postures to avoid diplopia. Additionally, most patients are not concerned about small vertical diplopia angles; the fusional amplitude is narrower on vertical deviation than on horizontal deviation. Ongoing inflammation and elastic changes in EOMs increase the numbers of affected muscles; binocular diplopia then spreads to the primary position, as well as the downward and horizontal gazes (115).

Usually, surgery is necessary to reduce GO diplopia when both the condition and the motility pattern have been stable for ≥ 6 months (116, 117). Coats et al. (118) explored whether strabismus surgery during active GO aided selected patients; they reported good surgical outcomes in eight patients whose parameters were stable for shorter times than suggested above.

Surgery seeks to create the largest possible binocular single vision fields, particularly in the primary and reading positions (115, 116). In recent decades, success has been graded as excellent, good, acceptable, and poor (119); a tool quantifying residual diplopia and the disease-specific quality-of-life has been developed (the GO-QOL) (120).

The muscles affected by GO are extremely tight. Considering the severe inflammation and thus the enhanced restriction of muscles that are already strongly contracted, recession of tight muscles is strongly recommended (121). Lee et al. (122) reported good surgical outcomes after vertical rectus resection in patients with large angles of deviation (≥ 20 prism diopter [PD]). Only normal-sized muscles were manipulated to prevent inflammation and adverse surgical outcomes. The most controversial issue in this field involves the decision to use (or not use) adjustable sutures. In procedures involving adjustable sutures, overcorrection was evident when recessing the IR muscle (123, 124). However, other studies have not revealed significant differences between fixed and adjustable sutures (125). No randomized controlled trials have compared adjustable and nonadjustable sutures. Kushner (126) reported that semi-adjustable suturing completely abolished muscle slippage. Although Jefferis et al. (127) adjusted recessed SR muscles, this method was only used in patients with complex restrictive disease or small vertical prism fusion ranges. The overcorrection of IR recession via postoperative drift is common (123, 128); it can be explained by impaired contralateral elevation and underestimation of the increased SR tone (129). Suggested approaches to mitigate the risk of consecutive hypertropia include planned surgical dosage reduction; a semi-adjustable hang-back approach toward large recessions; and a long horizontal, intrascleral simple hang-back for small recessions (130). Although bilateral MR recession is frequently used to correct horizontal diplopia, undercorrection may be associated with residual diplopia because the muscles are tight.

Strabismus surgery for GO patients is difficult; the outcomes are unpredictable. Plager (131) found that larger-than-expected recessions were necessary to treat small deviations and smaller-than-expected recessions were required when treating large GO-associated deviations; surgeons must carefully consider the deviations. Generally, the recessions are 3-4 PD/mm for the IR and 3-5 PD/mm for the MR (116, 130). Preoperative forced duction tests in all directions are useful. Nguyen et al. reported that a tailored plan addressed duction restriction; forced duction tests improved surgical success. The unpredictable outcomes of squint surgery for GO patients can mainly be attributed to EOM restriction; preoperative measurement of target muscle tension is critical. After IR recession, an A- or V-pattern deviation may appear if the adduction power is weakened. During reattachment, the recessed muscle should be moved in a nasal direction to reduce the risk of pattern deviation (112).

The success rates vary from 57% to 86% after initial surgery (124, 127, 128), depending on the success criteria used and the involved muscles (132). After orbital decompression surgery, poor prognostic factors include a severe restrictive pattern and a large deviation angle. Relative orbitopathy symmetry at onset and a shorter time between onset and surgery are factors predictive of good outcomes (133). In a recent study of 448 patients who underwent strabismus surgery, approximately 1 in 4 required reoperations; these mainly included patients in whom multiple muscles were involved during the initial surgery (134).

Vertical rectus muscle recession may exacerbate the retraction of both upper and lower lids. A preferred approach comprises the division of fibrous connections between the SR and upper lid levator complex, and between the IR and the lower lid retractors (112). Several approaches have been used for these purposes, including suturing of the desired point of eyelid retractor apposition to the recessed IR (135), separate suturing for postoperative adjustment of eyelid position (136), and sharp dissection of the fascia of the capsulopalpebral head combined with lysis of the fascial connections between the lower eyelid and the IR (137). Conversely, the recession of a tight IR can alleviate ipsilateral upper lid retraction by elevating the SR tone, consistent with Hering’s law.

New surgical techniques have been suggested in recent decades. Dal Canto et al. (138) described a unique approach for intraoperative determination of the position of rectus muscle reattachment. The cited authors allowed the disinserted muscle to rest on the eyeball sclera in the primary position. Such “intraoperative relaxed muscle positioning” considers muscle tightness; it has been associated with a good surgical success rate (88%) (138, 139). Other groups have also reported satisfactory results (140, 141). To reduce IR muscle restriction, tendon elongation uses homologous scleral grafts, polytetrafluoroethylene (Goretex), silicone, bovine pericardium, (142), or fascia lata (143). Jefferis et al. (127) reported favorable outcomes after prioritizing downgaze alignment (rather than primary gaze alignment) to avoid downgaze diplopia.

Postoperative complications after squint surgery include conjunctival injection and scarring, corneal dellen, pyogenic granuloma, muscle slippage and loss, pulled-in-two syndrome, periorbital and orbital cellulitis, scleral perforation, retinal detachment, endophthalmitis, anterior segment ischemia, and recurrent or consecutive postoperative diplopia (144–147). Changes in eyelid position and/or eyelid retraction can also occur, particularly if adjustable sutures are placed. Necrotizing scleritis may develop in patients with immune disorders (148). Because the muscles are extremely tight and the inflammation is pronounced, such complications may be more common if adjustable sutures are placed, compared with routine squint surgery. Such complications may be avoided by gentle manipulation during surgery, meticulous dissection from adjacent tissues including the capsulopalpebral head, and appropriate planning of adjustable suture positions.

## Lid surgery

Upper eyelid retraction, known as Dalrymple’s sign, is associated with a widened palpebral fissure. The British ophthalmologist John Dalrymple was the first to distinguish lid retraction from exophthalmos, based on the notion that the levator palpebrae superioris muscle can cause upper eyelid retraction (149). Lid retraction, particularly involving the upper eyelid, is the most common sign in GO patients (up to 90%) (150, 151). Because an abnormal lid position can expose the cornea and conjunctiva, ocular surface diseases (e.g., dry eye and exposure keratitis) may develop. Affected patients principally report ocular discomfort and poor cosmesis. Upper eyelid retraction is diagnosed when the lid margin is higher than the normal position of the upper lid (i.e., 1-2 mm below the upper limbus). Lower eyelid retraction is diagnosed if the lower sclera is visible—the normal position is the lower limbus.

Upper eyelid retraction reflects the contraction and fibrosis of levator and Mueller muscles (152, 153). Patients with GD typically exhibit increased sympathetic tone of the Mueller muscle, triggering upper eyelid retraction. Although lower eyelid retraction in GO patients has received less attention than upper eyelid retraction in such patients, Bartley et al. found that 85% of 120 GO patients exhibited lower eyelid retraction at diagnosis (154). Increased adrenergic stimulation of the inferior tarsal muscle, similar to Mueller muscle hyperaction in the upper eyelid, was among the initial theories proposed to explain lower eyelid retraction (155). Anatomically, lower eyelid retraction can be caused by fibrosis of the capsulopalpebral fascia and/or enlargement of the IR muscle (155).

The peak of the normal upper eyelid lies medial to the center of the pupil. However, in GO patients with upper eyelid retraction, the peak of the normal lid contour is lost; the upper lid continues to rise laterally. Thus, a temporal (or lateral) flare is characteristically observed. Additionally, lid lag may be evident when looking down; this constitutes von Graefe’s sign. Eyedrops with an adrenergic blocking agent (guanethidine) or a β-adrenergic blocking agent (propranolol) were previously used to treat mild lid retraction (156, 157); however, they are no longer preferred because of adverse effects including vasodilatation, irritation, and ocular discomfort (158–160).

Several reports have shown that lid retraction improves after Botox A injection into the skin or subconjunctiva of the upper lid. This injection method serves as a temporary treatment, both in the acute phase and prior to surgery. However, Botox A injection has been associated with limited transient ptosis and diplopia in GO patients with upper eyelid retraction (161, 162). Furthermore, subconjunctival triamcinolone acetonide (TA) injection can improve upper eyelid retraction in GO patients in the acute and active phases, but its adverse effects include temporary increases in intraocular pressure and ptosis (163–165). Subconjunctival TA injection is commonly administered as follows (163, 166, 167). An ice pack is used to cool the upper eyelid for 1 min to reduce pain and bleeding. Under downgaze, the upper eyelid skin is pulled upward into the supine position. After confirmation that blood reflux is absent, a needle is carefully inserted to a depth of approximately 1 cm. Generally, 0.5 mL (40 mg/mL) of subconjunctival TA is gently injected toward the orbital fat around the levator muscle. The results of several studies have suggested that this approach significantly decreases inflammation of the levator muscle and eyelid fat (163, 166, 167). However, symptom relief was less effective for GO cases with severe retractions. Additionally, intraocular pressure increased after steroid injection into the upper eyelid.

In GO patients, the most common causes of eyelid surgery are corneal and conjunctival exposure on lid retraction and a poor cosmetic appearance. Eyelid retraction surgery remains highly individualized. Its functional goals comprise the treatment of dryness, exposure keratitis, and lagophthalmos by lowering the upper eyelid margin to the normal position. Its esthetic goal comprises ensuring that the heights of the upper eyelid margins are natural and symmetrical. Most oculoplastic surgeons agree that, unless emergency surgery is required to treat medically uncontrollable exposure keratitis, the eyelid position should be stable for 6 to 12 months prior to surgery (168). A surgical decision is made after careful consultation with the patient, considering subjective symptoms and the objective ocular surface condition. Depending on severity, in mild cases, the management of upper eyelid retraction may be confined to conservative treatment with artificial tears (i.e., a nonsurgical method). Several studies showed that orbital decompression surgery could improve eyelid position and reduce proptosis. In patients who underwent consecutive medial and lateral orbital wall decompression, upper and lower lid retraction were improved. The extent of proptosis reduction was significantly associated with the level of lower lid retraction after surgery (169–171). One study investigated the correlation between the extent of enophthalmos and the interpalpebral fissure status of patients with unilateral orbital wall fractures; it revealed that patients with more severe enophthalmos tended to have fewer interpalpebral fissures (172).

Upper eyelid retraction surgery can be performed through a skin or conjunctival approach. An advantage of the skin approach is that anatomical structures inside the eyelids can be directly viewed. However, its disadvantages include a long operation time and extensive dissection. The conjunctival approach is shorter, but it is difficult to distinguish anatomical structures if the surgeon is unfamiliar with the operation.

The treatment of eyelid retraction via disinsertion of the levator and Mueller muscles was described by Henderson in 1965 (173). Since that time, several techniques have been introduced to correct upper eyelid retraction. These techniques involve anterior or posterior Muellerectomy with or without graded levator muscle disinsertion, the use of hang-back sutures, scleral interposition, and full-thickness eyelid transection (blepharotomy) (174–178). Although many studies have focused on the correction of upper eyelid retraction, no method has been strongly recommended. Randomized controlled trials comparing procedures are absent, and no approach is considered superior to others.

Similar to the method used for the upper eyelids, the correction of lower eyelid retraction can be performed through an anterior or posterior approach. Recession or extirpation of the capsulopalpebral fascia with or without spacer placement has been used to correct lower eyelid retraction. The spacer is a supportive material that elevates and maintains the eyelid against the force of gravity. A spacer was first used to correct lower lid retraction by Blair in the 1940s (179). Typical spacers include homologous sclera and tarsus; autologous hard palate mucosa, tarsal conjunctiva, cartilage, and dermis; and bioengineered matrices such as acellular dermis (AlloDerm), a porcine skin xenograft (Enduragen), porous polyethylene (Medpor), and a polyester mesh (Mersilene). All of these spacers are alloplastic materials that lack immunogenicity (155, 180). A few authors have compared grafts (181–183). A retrospective study in 2011 showed that, compared with a dermis-fat graft, AlloDerm was more effective in terms of lower eyelid retraction correction; however, the difference was not statistically significant (183). It is difficult to identify an optimal procedure or spacer because the investigations have not been standardized.

Conventionally, orbital decompression is performed first, followed by lid retraction. However, many studies in recent decades have shown that maximum correction is obtained when upper or lower lid retraction and orbital compression are conducted during a single procedure (184–187). One comparative clinical study investigated the outcomes of upper eyelid retraction surgery performed at or after the time of orbital decompression; transconjunctival Mueller muscle recession performed during deep lateral wall decompression yielded satisfactory results in 67% of 97 cases (187). Another comparative study compared the surgical outcomes of acellular human dermis grafting and lower eyelid retractor recession during orbital decompression; correction of eyelid retraction using the graft during orbital decompression provided excellent results (185).

No definitive treatment has been established for lid retraction in GO patients. Furthermore, it is difficult to predict the outcome and prognosis. Similar to the outcomes of other eyelid surgeries, overcorrection, undercorrection, and lid crease asymmetry may be evident after eyelid retraction. Undercorrection of either the temporal portion of the eyelid or the entire lid is a particularly common postoperative complication of Mueller muscle recession (188). Adequate lysis of the lateral horn of the levator aponeurosis and the Mueller muscle is recommended to avoid uncorrected lateral upper eyelid flare. Recommendations often include the graded levator hinge procedure or graded full-thickness blepharotomy (189–191).

In both the active and inflammatory phases, functional thyroid correction should be medically prioritized. Botox A and TA injections can be administered around the conjunctiva and eyelids in patients who exhibit severe dry eye disease and superficial keratitis caused by lid retraction. Moreover, for patients undergoing orbital decompression, concurrent correction of any lower lid retraction is recommended, using a spacer such as acellular dermal matrix. Importantly, GO patients who require surgery must be continuously monitored to detect anterior segment conditions such as dry eye disease and superficial keratitis.

## Conclusions

Rehabilitative surgery is usually included in the treatment of moderate-to-severe ophthalmopathy during the inactive phase; it is intended to reduce proptosis, restore function, and enhance appearance. Typically, the constituent surgical procedures are performed in a fixed sequence, commencing with orbital decompression. Although many approaches to surgical decompression have been optimized, few controlled studies have been conducted regarding their relative efficacies. Therefore, the key objective of surgery and the surgeon’s skills are primary concerns when choosing an approach. In diplopia patients, surgical decompression generally precedes strabismus surgery that corrects eye motility abnormalities. Other functional and cosmetic issues are managed later; these issues include facelifting, soft tissue filler injection, and eyelid repair.

Even the use of advanced surgical techniques by the growing number of well-trained surgeons can never fully avoid mild-to-severe complications. There is a need to continue clinical and laboratory investigations of new drugs that reduce long-term orbit deformity, alleviate the requirement for rehabilitative surgery, and improve long-term quality-of-life.

## Author contributions

JB, HSC, CK, HK, and SYJ wrote the first draft of the manuscript. JB and SYJ contributed to conception. SYJ reviewed and edited the mansucript. All authors contributed to manuscript revision, read, and approved the submitted version
